# Research progress on the STAT signaling pathway in pregnancy and pregnancy-associated disorders

**DOI:** 10.3389/fimmu.2023.1331964

**Published:** 2024-01-03

**Authors:** Lihua Li, Zhen Zhang, Haoyang Li, Miaomiao Zhou, Fang Li, Chu Chu, Yunhong Zhang, Xiaoxiao Zhu, Hongmei Ju, Xia Li

**Affiliations:** ^1^ Innovative Institute of Chinese Medicine and Pharmacy, Shandong University of Traditional Chinese Medicine, Jinan, China; ^2^ School of Clinical and Basic Medical Sciences, Shandong First Medical University and Shandong Academy of Medical Sciences, Jinan, Shandong, China; ^3^ International Business School, Tianjin Foreign Studies University, Tianjin, China; ^4^ Affiliated Hospital of Shandong University of Traditional Chinese Medicine, Jinan, Shandong, China

**Keywords:** signal transducer and activator of transcription, pregnancy, pregnancy-related disorders, maternal-fetal interface, maternal-fetal immune tolerance

## Abstract

Signal transducer and activator of transcription (STAT) proteins, pivotal regulators of signaling cascades, undergo activation in response to the stimulation of cytokines and growth factors, and participate in biological processes, including inflammation, immune responses, cell proliferation, and differentiation. During the process of pregnancy, STAT signaling is involved in regulating embryonic implantation, endometrial decidualization, and establishing and maintaining maternal-fetal immune tolerance. Increasing evidence suggests that aberrant STAT signaling contributes to the occurrence and development of pregnancy disorders, including repeated implantation failure (RIF), preeclampsia (PE), recurrent spontaneous abortion (RSA), preterm birth (PTB) and gestational diabetes mellitus (GDM). Elucidating the molecular mechanisms of the STAT signaling pathway holds promise for further understanding the establishment and maintenance of normal pregnancy, and thereby providing potent targets and strategic avenues for the prevention and management of ailments associated with pregnancy. In this review, we summarized the roles of the STAT signaling pathway and its related regulatory function in embryonic implantation, endometrial decidualization, and maternal-fetal immune tolerance. In conclusion, in-depth research on the mechanism of the STAT signaling pathway not only enhances our understanding of normal pregnancy processes but also offers STAT-based therapeutic approaches to protect women from the burden of pregnancy-related disorders.

## 1 Introduction

Pregnancy refers to the process of normal growth and development of the embryo within the mother’s uterus. Normal pregnancy relies on successful embryo implantation, endometrial decidualization, placental formation, and immune balance at the maternal-fetal interface. In early pregnancy, trophoblast cells proliferate and implant into the endometrium, while endometrial decidualization increases endometrial receptivity, facilitating embryo implantation (1). As the embryo carries paternal genetic information and is considered a semi-allograft similar to successful organ transplantation, the development and preservation of maternal-fetal immune tolerance are crucial for embryo implantation and development. During this process, the immune mechanisms of the organism play a complex and precise regulatory role (2). Therefore, exploring the regulatory mechanisms of various aspects of the maternal-fetal interface will further elucidate the mechanisms underlying the establishment and maintenance of successful pregnancy, presenting innovative targets and insights for the prevention and management of pregnancy-associated disorders.

Signal transducer and activator of transcription (STAT) proteins are DNA-binding proteins that participate in signal transduction and control of gene transcription. The STAT protein family comprises seven members, namely STAT1, STAT2, STAT3, STAT4, STAT5A, STAT5B, and STAT6. Structurally, STATs consist of an amino-terminal domain, a coiled-coil domain, a DNA-binding domain, an SH2 domain and a carboxy-terminal transactivation domain (3). The amino-terminal domain facilitates the dimerization of STATs, the DNA binding domain discriminates the DNA motifs, and the SH2 domain recognizes the phosphorylated tyrosine on the intracellular domain of the receptors. They can be activated by extracellular molecules, especially hormones, and cytokines, including interferons (IFNs), interleukins (ILs), and growth factors. Upon binding to the corresponding transmembrane receptors of such extracellular ligands, Janus kinases will be recruited and activated, which then phosphorylate tyrosine residues on the receptor’s catalytic domain. This further leads to the recruitment and phosphorylation of STAT proteins. Phosphorylated STAT proteins form homo- or heterodimers upon activation and subsequently translocate into the nucleus, where they function as regulators of gene transcription to regulate biological and pathological processes (4). Finally, STATs undergo dephosphorylation in the nucleus and returned to the cytosol to terminal the signal and keep homeostasis of related cells.

The STAT signaling pathways has been found to govern various cellular processes including proliferation, differentiation, and migration (5). Growing body of research suggests that the STAT signaling pathway regulates endometrial stromal cell decidualization, trophoblast proliferation and implantation, spiral artery remodeling, and maternal-fetal immune tolerance. Aberrations in the STAT signaling pathway have been implicated in the pathogenesis of diverse pregnancy-related disorders (6). Therefore, in-depth research on the STAT signaling pathway is of significant importance for understanding normal pregnancy establishment and the prevention and treatment of pregnancy-related disorders.

## 2 The function of the STAT signaling pathways in normal pregnancy

### 2.1 Regulation of endometrial decidualization and proliferation by the STAT signaling pathways

**Figure 1:**
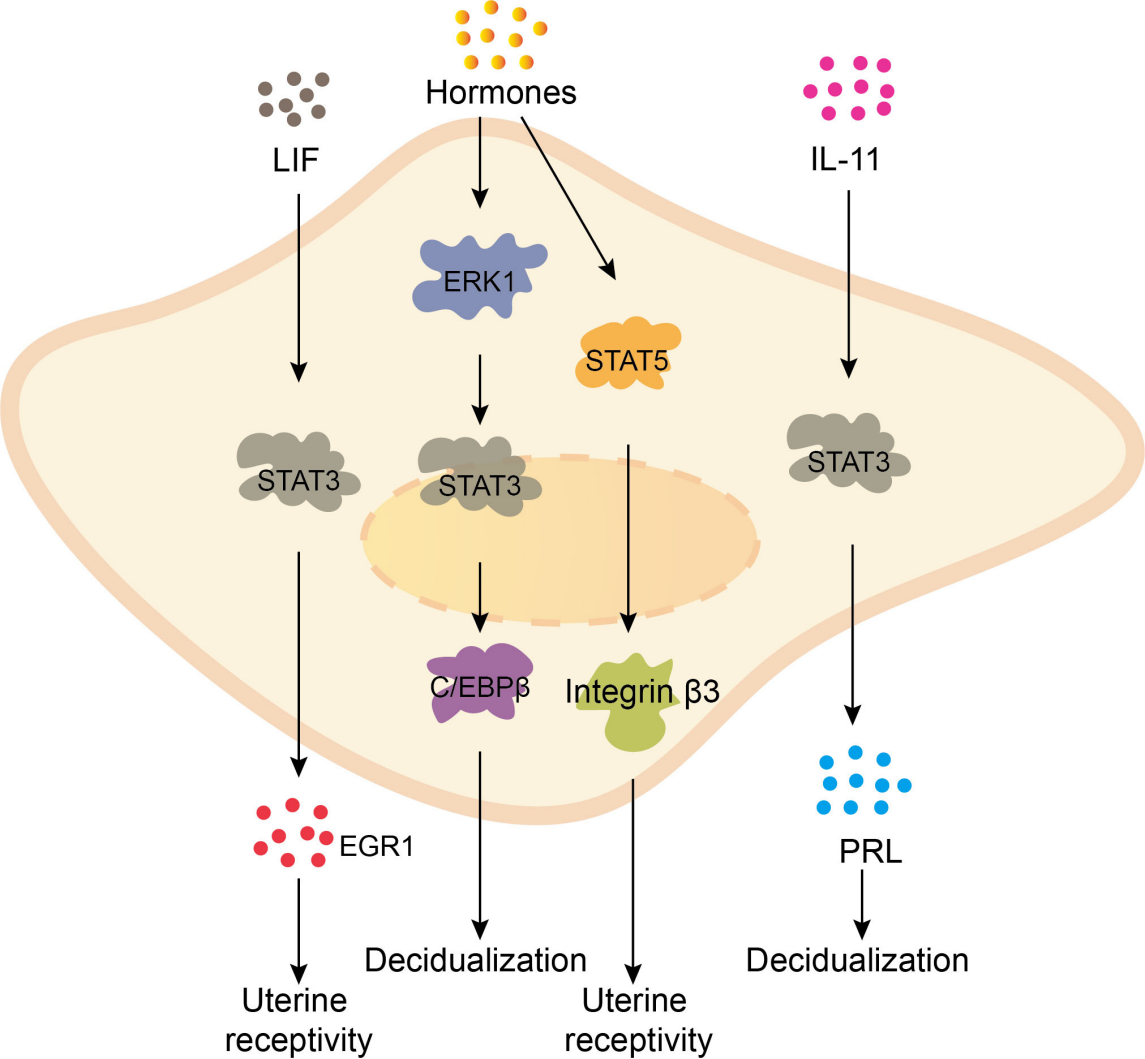
The STAT signaling pathway promotes the decidualization of the endometrium. LIF promotes the expression of EGR1 through stimulating STAT3 signaling pathway, enhancing uterine receptivity. Hormones upregulate the expression of transcription factor C/EBPβ by activating ERK1/STAT3 pathway in endometrial stromal cells, promoting endometrial decidualization. Besides, hormones activate the JAK2/STAT5 pathway in decidual cells, increasing the expression of receptivity-related gene integrin β3 and improving uterine receptivity. IL-11 activates STAT3 in decidual cells, promoting the expression of PRL, accelerating the process of decidualization.

The process of endometrial decidualization involves the activation of multiple signaling pathways of the uterine endometrial stromal cells, stimulated by hormones, growth factors, and other factors. This stimulation prompts the proliferation and differentiation of the endometrial cells into enlarged, rounded, cytoplasm-rich, and multinucleated decidual cells. The normal expansion and differentiation of endometrial cells are crucial for successful embryo implantation (7). Research has revealed that the STAT signaling pathway plays a significant role in the processes of decidual cell proliferation and differentiation. During early pregnancy, the hormones, including estrogen and progesterone, activate the ERK1/STAT3 signaling pathway in the endometrial stroma, thereby promoting the expression of the transcription factor CCAAT/enhancer binding protein β (C/EBPβ) and stimulating decidualization of human endometrial stromal cells (8, 9). Simultaneously, decidual cells secrete prolactin (PRL), which facilitates the progression of decidualization. Besides, PRL secretion levels can serve as an indicator of the degree of decidual cell differentiation (10). Interleukin-6 (IL-6) and -11 (IL-11) can promote endometrial decidualization through the activation of STAT3 signaling pathway, with IL-11 primarily mediating this process through increased PRL expression (11, 12). Additionally, leukemia inhibitory factor (LIF) triggers the activation of STAT3 signaling pathway in decidual cells, promoting the expression of Early Growth Response 1 (EGR1) and enhancing uterine receptivity (13). Moreover, placental growth hormone (GH) acts on the JAK2/STAT5 pathway in decidual cells, increasing the expression of integrin β3, which is associated with receptivity, and promoting uterine receptivity for pregnancy (14). Furthermore, high levels of hormonal stimulation may induce increased STAT5 expression, subsequently promoting the activation of the PRL promoter and facilitating endometrial decidualization (15). In summary, various cytokines and hormones activate the STAT signaling pathway within decidual cells, regulating cell proliferation and differentiation to promote endometrial decidualization and maintain a healthy pregnancy (**Figure 1**).

### 2.2 Regulation of trophoblast cell function by the STAT signaling pathways

**Figure 2:**
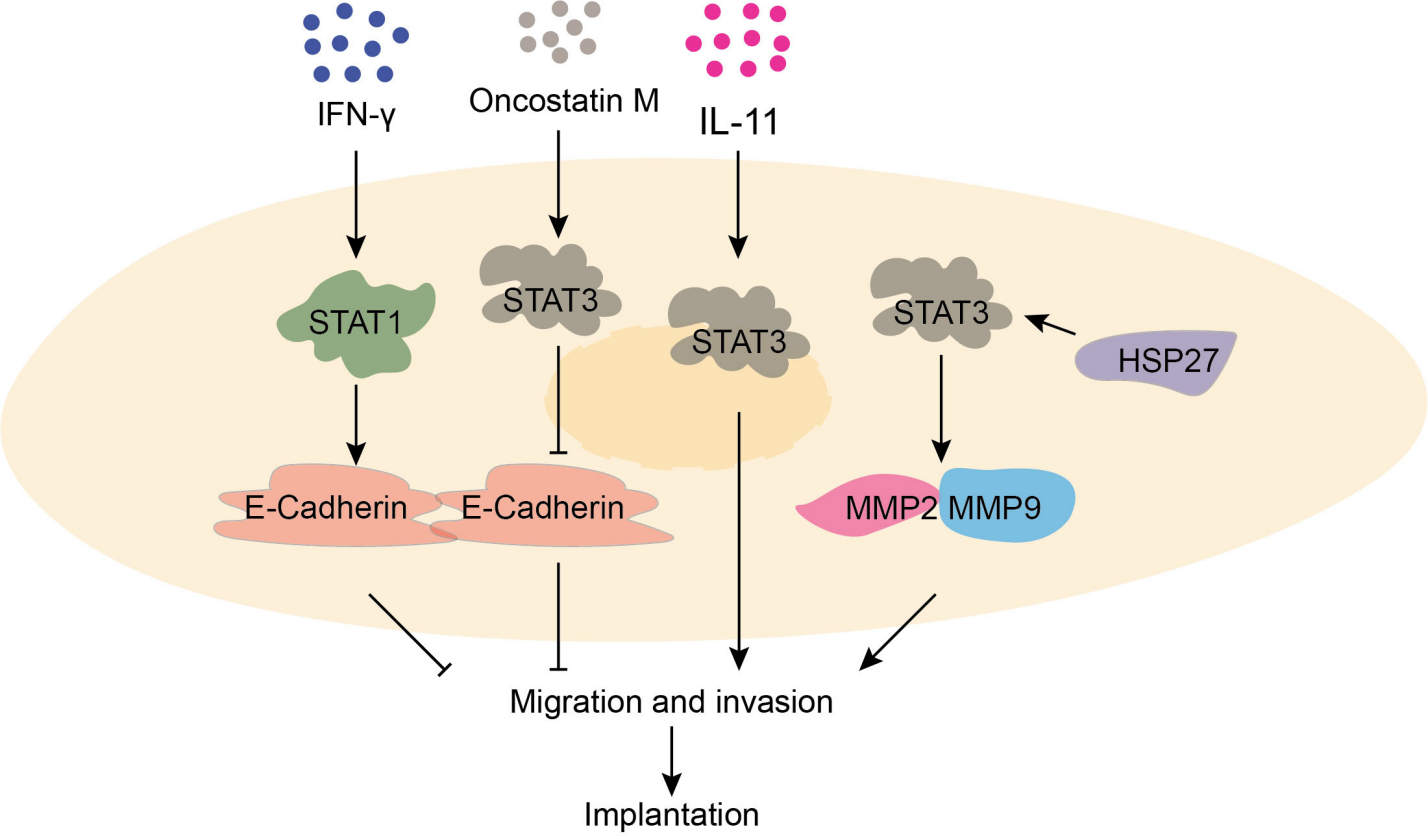
The function of STAT signaling pathway in trophoblast invasion and migration is critical for normal pregnancy. IFN-γ prompts the expression of E-cadherin by activating the STAT1 signaling pathway, which prevents excessive cell migration. OSM promotes the expression of MMP2 and MMP9 by activating the STAT3 signaling pathway, which promotes migration. IL-11 promotes cell invasion and migration by activating STAT3 signaling pathway. HSP27 promotes the expression of MMP2 and MMP9 by activating the STAT3 signaling pathway.

Trophoblast cells refer to non-embryonic cells with nourishing functions. Research has demonstrated that the STAT signaling pathway is involved in the regulation of various processes such as trophoblast cell implantation and spiral artery remodeling. During early pregnancy, moderate level of interferon γ (IFN-γ) activates JAK/STAT1 signaling pathway, promoting E-cadherin expression to prevent excessive migration and invasion of trophoblast cells. Additionally, IFN-γ stimulates trophoblast cells to secrete vascular endothelial growth factor (VEGF-C), which aids in spiral artery remodeling (16, 17). Furthermore, in the chorionic tissue and serum of early pregnant women, high levels of IL-11 and oncostatin M (OSM) have been observed. These molecules activate the STAT3 signaling pathway of trophoblast cells, enhancing Matrix Metallopeptidase 2 (MMP2) and 9 (MMP9) expression while suppressing E-cadherin expression, thereby promoting trophoblast cell implantation (18, 19). Moreover, studies have found that STAT3 signaling pathway can be activated by heat shock protein-27, leading to increased expression of MMP2 and MMP9 and facilitating cell implantation of trophoblast cells (20). In summary, the regulation of trophoblast cell function by the STAT signaling pathway plays critical roles in normal pregnancy (**Figure 2**).

### 2.3 Regulation of the maternal-fetal interface immune response by the STAT signaling pathways

**Figure 3:**
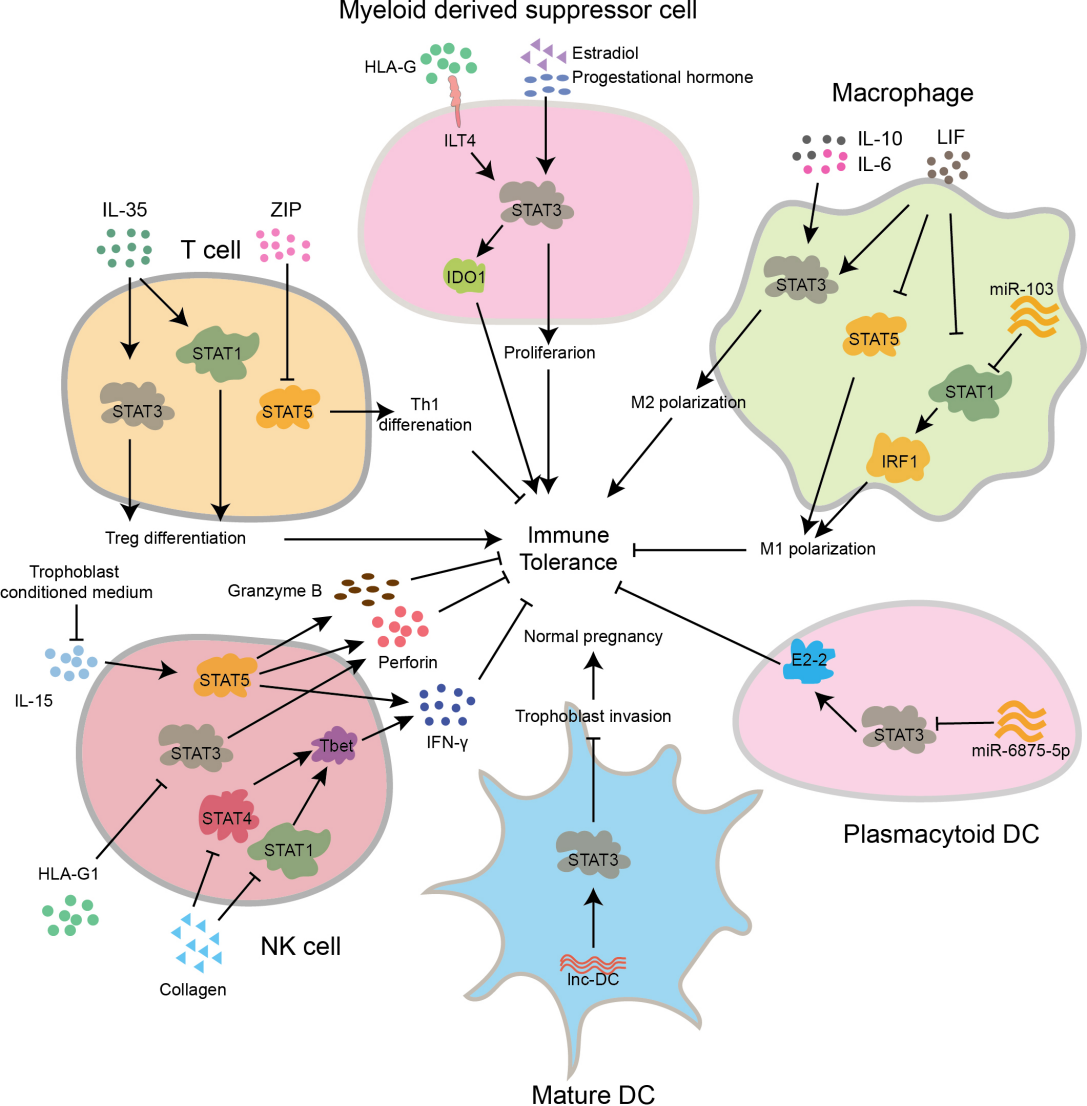
The STAT signaling pathway regulates the differentiation and function of immune cells, maintaining normal pregnancy. In early pregnancy, IL-35 activates STAT1 and STAT3 to promote Treg differentiation, while ZIP inhibits STAT5 activation to promote Th1 differentiation. In myeloid derived suppressor cell of first trimester, HLA-G binds to the receptor ILT4 to activate STAT3, promoting the secretion of IDO1 and inducing immune tolerance. Meanwhile, estradiol and progestational hormone can also promote its proliferation through activation of STAT3. In macrophages of early gestation, IL-6, IL-10, and LIF activate STAT3 to promote Mφ2 polarization, while LIF can also inhibit STAT1 and STAT5 to inhibit Mφ1 polarization. Besides, miR-103 inhibits Mφ1 polarization by suppressing STAT1 and IRF1. In pDC of first pregnancy stage, miR-6875-5p inhibits STAT3 and E2-2, leading to a suppression of immune tolerance. In mature DCs, lnc-DC activates STAT3, suppresses the invasion capability of trophoblast cells, and thus inhibits normal pregnancy. In decidual NK cells of human primary first-trimester, collagen suppresses STAT1 and STAT4, inhibiting the excessive expression of T-bet and the secretion of IFN-γ., In late stage of pregnancy, HLAG1 inhibits STAT3 activation, leading to suppressed secretion of perforin. In pregnancy early, inhibiting the activation pathway induced by IL-15 can reduce the secretion of Granzyme B, perforin and IFN-γ. All of the above factors contribute to immune tolerance.

During pregnancy, the immune cells found at the interface between mother and fetus include decidual natural killer cells (dNK), decidual macrophages (dMφ), decidual T cells (dT), and decidual dendritic cells (dDC). These immune cells play crucial roles in regulating immune tolerance (21, 22). The STAT signaling pathways participate in maintaining the balance of immune tolerance by regulating the functions of these immune cells, which is essential for a successful pregnancy (**Figure 3**).

#### 2.3.1 Regulation of NK cell differentiation and activity by STAT

dNK cells, accounting for 70% of decidual immune cells, are mainly composed of CD56^+^/CD16^-^ NK cells. They possess unique surface receptors and can induce immune tolerance through the secretion of granules (23, 24). Research has indicated that the JAK/STAT signaling pathway plays a role in controlling the development and function of NK cells (25). In human primary first-trimester decidual tissue, decidual cells bind to collagen proteins on the surface of dNK cells, inhibiting the activation of JAK1, JAK2, and downstream STAT1 and STAT4, thereby suppressing the expression of T-bet, reducing transcription of IFN-γ of dNK cells, and inducing maternal-fetal immune tolerance (26). In addition, human leukocyte antigen-G1 (HLA-G1) inhibits the STAT3 signaling pathway in dNK cells, reduces perforin expression, and induces immune tolerance (27). Furthermore, conditioned medium of trophoblast cells suppressed IL-15 induced JAK3/STAT5 signaling pathway in dNK cells, leading to decreased expression of perforin, granzyme B, and IFN-γ, and thus favoring pregnancy (28). In summary, STAT-mediated signaling pathways regulate NK cell function and induce immune tolerance.

#### 2.3.2 Regulation of macrophage differentiation and function by STAT

dMφ cells, accounting for 20% of decidual immune cells, mainly consist of M1-type (dMφ1) and M2-type (dMφ2) decidual macrophages. dM1 cells secrete pro-inflammatory cytokines like TNF-α, IL-12, IL-23, as well as reactive oxygen species (ROS), while dM2 cells primarily secrete regulatory cytokines such as IL-10 and transforming growth factor beta1 (TGF-β1) (29, 30). Recent studies also revealed that the STAT signaling pathways play pivotal roles in dMφ differentiation. Compared to the abortion group, dMφ isolated from normal pregnant mice show increased expression of B7-H4, which mediates inhibition of M1 polarization through suppression of the JAK2/STAT1 signaling pathway (31). Besides, elevated expression of miR-103 in human dMφ cells can inhibit the differentiation of M1 macrophages and induce immune tolerance by interacting with the non-coding region of STAT1 and suppressing the expression of transcription factor IRF1 (32). It has also been found that IL-6 secreted by trophoblasts is capable of activating the STAT3 signaling pathway in dMφ cells, thereby promoting M2 polarization and induce immunosuppression (33, 34). Additionally, IL-10 derived from trophoblasts activates dMφ cells via JAK/STAT3 signaling, inducing M2 macrophage differentiation and protecting pregnancy (35). Moreover, LIF secreted by dNK cells inhibits IFNγ/STAT1 and GM-CSF/STAT5, while activating STAT3 through binding to surface receptors on dMφ cells, thereby reducing inflammation mediated by M1 macrophages during pregnancy (36). In conclusion, STAT-mediated signaling pathways are involved in macrophage polarization and functional regulation, playing a significant role in preserving immune tolerance at the interface between the mother and fetus.

#### 2.3.3 The role of STAT-mediated regulation of helper T cell differentiation and function in pregnancy

The dynamic equilibrium of the adaptive immune system allows for an immune response against invading pathogens while maintaining tolerance toward the semi-allogeneic fetus, which is crucial for successful pregnancy. Helper T (Th) cells comprise mainly of Th1, Th2, Th17, and regulatory T cells (Treg). The STAT signaling pathways play vital roles in maintaining immune balance during pregnancy by regulating the differentiation of helper T cells through the modulation of the expression of transcription factors (37). It has been revealed that IL-35 derived from trophoblast cells activates STAT1 and STAT3 through binding to T cell surface receptors, which subsequently inhibits conventional T cell proliferation and facilitates the initial differentiation of T cells into regulatory T cells, thereby inducing immune tolerance (38). Researchers have also found that the addition of interleukin-23 (IL-23) to extracted human decidual immune cells increases the proportion of Th17 cells and induces inflammation by activating the STAT3 signaling pathway. Conversely, the addition of IL-23 antibodies inhibits STAT3 activation, increases the proportion of Treg cells, promotes IL-10 expression, and induces immune tolerance (39). Furthermore, IL-2 can activate the JAK/STAT5 signaling pathway by binding to receptors on T cells, leading to FOXP3 expression and promoting Treg differentiation (40). Additionally, high levels of zeta inhibitory peptide (ZIP) in the peripheral blood of early-stage pregnant women can reduce Th1 cell polarization by inhibiting the JAK3/STAT5 signaling pathway, which helps in reducing of pro-inflammatory cytokines, and inducing immune tolerance to support normal pregnancy (41). In summary, the STAT signaling pathways play crucial roles in governing the differentiation and function of T cell subsets in maintaining maternal-fetal immune tolerance. However, the specific regulatory mechanisms are not fully understood and require further exploration.

#### 2.3.4 The modulation of dendritic cell differentiation and function by STAT

Accumulative evidence has shown that the STAT signaling pathways also participate in regulating the differentiation and function of dendritic cell (DC) subsets during pregnancy establishment and maintenance. In dDCs, it has been found that downregulation of miR-6875-5p activates the STAT3/E2-2 pathway, promoting the differentiation of plasma-like dendritic cell (pDCs), which is crucial for normal pregnancy (42). Besides, *in vitro* experiments have demonstrated that inhibition on lnc-DC in mature DCs reduces STAT3 activity, inhibits the expression of tissue inhibitor of metalloproteinase-1 (TIMP1) and -2 (TIMP2), but enhances MMP9 and MMP2 to ensure normal pregnancy progression (43–45). Furthermore, inhibition of the STAT5/ID2 signaling pathway in decidual tissue hinders the differentiation of classical DCs and induces maternal-fetal immune tolerance (46). Overall, the regulatory role of STAT in DC function is advantageous for preserving immune tolerance at the maternal-fetal interface during pregnancy.

#### 2.3.5 The regulation of myeloid-derived suppressor cells (MDSCs) differentiation and function by STAT

MDSCs, which originated from the myeloid lineage, are highly immunosuppressive and can be classified into polymorphonuclear MDSCs (PMN-MDSCs) and monocytic MDSCs (M-MDSCs). MDSCs express the receptor for HLA-G, known as immunoglobulin-like transcript 4 (ILT4). Research has demonstrated that HLA-G can activate STAT3 through ILT4, inducing the production of indoleamine 2,3-dioxygenase (IDO), which in turn promotes immune tolerance to maintain normal pregnancy (47, 48). In early pregnancy, elevated levels of estradiol and progesterone in women’s serum activate the ILT4/STAT3 signaling pathway to promote the expansion of MDSCs. Moreover, *in vitro* experiments have also found that decidual-derived IL-6 promotes the differentiation of peripheral blood neutrophils into PMN-MDSCs by stimulating STAT3 signaling pathway, thus contributing to immune tolerance (47, 49–51). Overall, STAT3 is critically involved in the regulation of MDSCs differentiation and contributes to the maintenance of immune tolerance.

## 3 The role of the STAT signaling pathway in pregnancy-related diseases

The STAT signaling pathways are important in establishing/maintaining maternal-fetal immune tolerance. Aberrant expression or over-activation of the STAT signaling pathway can lead to pregnancy-related diseases such as repeated implantation failure (RIF), preeclampsia (PE), recurrent spontaneous abortion (RSA), preterm birth (PTB), and gestational diabetes mellitus (GDM). Therefore, investigating the mechanisms by which the STAT signaling pathways contribute to pregnancy-related diseases can provide targets and evidence for the diagnosis and treatment of pregnancy-related diseases.

### 3.1 The role of the STAT signaling pathways in RIF

RIF refers to the failure of successful embryo implantation after multiple embryo transfers (52). Successful embryo implantation relies on embryo competence, endometrium receptivity, and immune tolerance at the interface between the embryo and the maternal environment. Studies have shown the involvement of STAT3 signaling pathway in RIF. For instance, it has been found that RIF patients exhibit decreased expression of LIF and STAT3 in endometrial cells, resulting in reduced uterine receptivity, and inhibiting embryo implantation. Studies in mouse models further confirmed that LIF/STAT3 signaling pathway inhibition contributes to embryo implantation failure (53–55). Moreover, it also suggests that downregulation of miR-30d-5p in endometrial cells of RIF patients can increase the level of suppressor of cytokine signaling 1 (SOCS1), which inhibits the LIF/STAT3 signaling pathway, and lead to failure of embryo implantation (56). Additionally, in endometrial cells of RIF patients, downregulation of progesterone-induced blocking factor 1 (PIBF1) inhibits the expression of IL-6, impedes STAT3 activation, reduces the expression of proliferation-related and decidualization-related genes, and ultimately disrupts the decidualization process (11). In conclusion, enhancing endometrial receptivity through decidualization is crucial for successful embryo implantation, and STAT3, as a key regulatory factor, could potentially be targeted therapeutically to treat RIF.

### 3.2 The role of the STAT signaling pathway in preeclampsia

Preeclampsia is a common, but severe pregnancy complication characterized by maternal high blood pressure, proteinuria, and endothelial dysfunction. Placental ischemia and hypoxia caused by impaired remodeling of trophoblast function are the main pathogenic causes for preeclampsia. The STAT signaling pathways regulate the functions of trophoblast cells and inflammatory responses involved in the development of preeclampsia. Therefore, deep understanding of STAT pathological roles can help prevent the occurrence and provide targets for the treatment of preeclampsia.

#### 3.2.1 The role of STAT in regulating cellular function in preeclampsia

The STAT signaling pathways primarily contribute to the development of preeclampsia by modulating the function of trophoblast cells. It has been reported that the expression of ribosomal protein S4 is elevated in placental tissues of patients with preeclampsia, while the phosphorylation level of STAT3 in serum is decreased. *In vitro* experiments have demonstrated that ribosomal protein S4 silencing up-regulates the expression of STAT3 in HTR8/SVneo trophoblast cells and increases the levels of N-cadherin and vimentin proteins, thereby promoting the invasion of trophoblast cells (57–59). Additionally, it has been discovered that down-regulation of Annexin7 in placenta of preeclampsia patients inhibits the JAK1/STAT3 pathway in trophoblast cells, leading to a decrease in BCL2 protein levels and induction of cell apoptosis, thus contributing to the development of preeclampsia (60). Moreover, increased expression of RAR-related orphan receptor A (RORA) has been observed in placenta of preeclampsia patients and HTR-8/SVneo cells. Suppression of RORA enhances the migration, invasion, epithelial-mesenchymal transition, proliferation, and angiogenesis of HTR-8/SVneo cells subjected to hypoxia treatment. Mechanistically, RORA activates the JAK2/STAT3 signaling pathway to exacerbate preeclampsia (61). Furthermore, it has been found that miR-125b is up-regulated in the serum of preeclampsia patients, which inhibits the STAT3 pathway and suppresses the migration and invasion of extravillous trophoblast cells (62). In summary, the suppression of the STAT3 signaling pathway in trophoblast cells may lead to cell apoptosis and impaired cell migration, thus facilitating the onset of preeclampsia.

#### 3.2.2 The role of STAT in regulating inflammation in preeclampsia

STAT can also participate in the development of preeclampsia by regulating inflammatory responses. Clinical data have revealed that nuclear factor of activated T cells-1 (NFAT-1), STAT1, and activator protein-1 (AP-1) were over-activated in monocytes of early-onset preeclampsia. Conversely, NFAT-1, STAT-1, and AP-1 are down-regulated in T cells of early-onset preeclampsia. This suggests that innate immunity is excessively activated while adaptive immunity is suppressed during the development of early-onset preeclampsia, and NFAT-1, STAT1, and AP-1 may serve as core transcription factors maintaining the equilibrium between innate and adaptive immune responses in the pathogenesis of early-onset preeclampsia (63). In addition, LIF can induce inflammation and endothelial dysfunction by increasing the expression of intercellular adhesion molecule-1 (ICAM-1) and vascular cell adhesion molecule-1 (VCAM-1) through the JAK/STAT3 pathway. Therefore, understanding the link between LIF and the pathogenic mechanisms of preeclampsia may contribute to the development of effective treatments for preeclampsia (64). Additionally, studies have shown that elevated expression of STAT4 in the serum of preeclampsia patients could act as a diagnostic indicator for the severity of the disease (65).

### 3.3 Research on the STAT signaling pathway in RSA

Clinically, the occurrence of two or more consecutive spontaneous abortions within 20 weeks of gestation is called recurrent spontaneous abortion (RSA). The causes for RSA pathogenesis are complex, and except for known factors such as chromosomal abnormalities, endocrine disorders, and uterine abnormalities, the etiology remains unclear for most RSA patients. Cumulative evidence has demonstrated that the STAT signaling pathways are involved in regulating the processes of trophoblast proliferation, implantation, spiral artery remodeling, and immune tolerance, which participates in the pathogenesis of RSA.

#### 3.3.1 The role of STAT in modulating trophoblast function of RSA

Multiple studies have demonstrated that abnormal expression of STAT in trophoblast cells contributes to cell proliferation and implantation dysfunction, resulting in the occurrence of diseases such as RSA. In early pregnancy, the expression of Sprouty 4 (SPRY4) is markedly increased in the trophoblast layer of RSA patients, accompanied by up-regulation of STAT1 and phosphorylated STAT1 (p-STAT1). Mechanistically, IFN-γ promotes SPRY4 expression and STAT1 phosphorylation through the PI3K/AKT pathway, thereby inhibiting trophoblast cell proliferation and accelerating apoptosis. So, elevated levels of SPRY4 and STAT1 potentially contribute to the onset and advancement of RSA, and could serve as targets for therapeutic intervention. Other studies have reported a substantial decrease in trophoblast cell numbers in early RSA patients, along with down-regulation of STAT3 and its downstream target genes cyclin D1 (CCND1) and vascular endothelial growth factor A (VEGF) in miscarriage tissues (chorionic villi and decidua). In addition, inhibition of the STAT3 signaling pathway *in vitro* can impede trophoblast cell growth and promote apoptosis (66–68). Furthermore, reduced expression of fascin in placental trophoblasts has been observed in early-stage RSA patients. Knockdown of fascin inhibits cell proliferation and increases apoptosis, which may be partially attributed to the down-regulation of STAT3 activity (69). Additionally, high expression of nerve injury-induced protein 1 (NINJ1) in villous tissues of RSA patients has been found to inhibit STAT3 activation in trophoblasts, leading to decreased cell proliferation, migration, and invasion, and ultimately resulting in RSA (70). Overall, both STAT1 and STAT3 affect cell migration through the regulation of genes associated with proliferation and migration, which is consistent with clinical observations. These findings provide a basis for further exploration of their feasibility as clinical targets.

#### 3.3.2 The role of STAT in regulating immune tolerance in RSA

The successful establishment of pregnancy relies on the immune tolerance balance maintained by various immune cells at the maternal-fetal interface. Research has shown that activation of the JAK2/STAT1 pathway in decidual natural killer (dNK) cells by IFN-γ significantly increases the expression of CX3CL1, inducing the homing of CD49b^+^ NK cells to the uterus and ultimately leading to RSA (71). Moreover, IL-6 and IL-23 activation of STAT3 in RSA patients promotes the expression of retinoic acid-related orphan receptor gamma T (RORγt), facilitating Th17 differentiation and the release of inflammatory factors while inhibiting the proliferation of decidual Treg cells, disrupting maternal-fetal immune tolerance and resulting in RSA (72, 73). In peripheral blood from RSA patients, downregulated expression of IL-2 inhibits STAT5 activation, thereby reducing FOXP3 expression and hindering Treg differentiation, disrupting immune balance and causing miscarriage (74). In peripheral blood natural killer cells (pNK), the IL-4/STAT6 signaling pathway promotes the surface expression of Tim3, facilitating the generation of anti-inflammatory cytokines and inducible regulatory Treg cells through a mechanism that depends on TGF-β1, which helps reduce the occurrence of miscarriage (72, 75). Furthermore, as formerly mentioned, dys-regulated miRs also causes RSA pathogenesis. For instance, diminished expression of miR-103 in decidual macrophages can promote M1 macrophage polarization by activating STAT1, while elevated expression of miR-6875-5p in decidual tissue inhibiting pDC differentiation and leading to RSA (32, 42). Overall, STATs play crucial roles in regulating the differentiation and function of immune cells, making it a key target for RSA treatment and prevention.

### 3.4 The role of the STAT signaling pathways in preterm birth

Preterm birth refers to premature delivery within 28 to 37 weeks of pregnancy. The occurrence of preterm birth is associated with excessive activation of inflammatory signaling pathways in maternal-fetal interface. Compared to women with normal pregnancies, elevated expression of IL-27 in the decidua tissue can bind to T-cell surface receptors, activating the JAK1/STAT1/STAT3 signaling pathway, promoting the expression of transcription factor T-bet, and enhancing the expression of chemokine 11 (CXCL11), chemokine 2 (CXCL2), and chemokine 1 (CXCL1), thereby facilitating the infiltration of Th1 cells into the decidua and leading to PTB (76). Additionally, the inflammatory cytokine IL-6 activates the JAK2/STAT3 signaling pathway in trophoblast cells, suppressing the expression of B-cell lymphoma-2 (BCL2), promoting the expression of BCL2-associated X (BAX), and apoptosis mediated PTB (77). In summary, effective interventions targeting Th1 cell response and inflammation are of significant importance for the prevention and management of PTB.

### 3.5 Research on the STAT signaling pathway in gestational diabetes mellitus

Gestational diabetes mellitus (GDM) is characterized by impaired glucose tolerance that occurs in the early stages of pregnancy. Studies on GDM mouse models have found that hepatocyte growth factor (HGF) can promote insulin secretion in pancreatic β cells by activating STAT5, while IL-6, IL-1β, and IL-33 inhibit insulin secretion by activating the JAK2/STAT3 signaling pathway, contributing to the development of the disease (78). Research on GDM rat models has shown that activation of the STAT1 and STAT5 signaling pathways in β cells participate in lowering sugar in blood (79, 80). Additionally, through testing the serum, placenta, and umbilical cord blood of GDM patients, researchers have found that elevated expression of STAT3 is involved in regulating metabolic pathways and the occurrence of gestational diabetes (81, 82). These studies provide a basis for exploring the pathogenesis and prevention/treatment of gestational diabetes.

## 4 The role of the STAT signaling pathways in the prevention and treatment of pregnancy-related disorders

Currently, some pharmaceutical drugs have been used to target the STAT protein-related signaling pathways for the treatment of pregnancy-related diseases (**Table 1**). Sulfasalazine inhibits STAT3 activation and induces the reduction of soluble Fms-like tyrosine kinase-1 (sFlt-1) and endothelial growth factor receptor-1 in placental tissue, thereby decreasing the risk of preeclampsia (87). Additionally, montelukast plays an anti-inflammatory and antioxidant role by inhibiting the JAK2/STAT3 signaling pathway in placental cells of preeclampsia mice, thereby improving the pathological condition of preeclampsia (86, 96). Moreover, in a preeclampsia rat model, silencing Annexin A1 in placental cells can inhibit the expression of TNF-α, IL-1β, IL-6, and IL-8 by inhibiting the JAK2/STAT3 signaling pathway. This can help alleviate inflammation and inhibit cell apoptosis by decreasing BAX levels and increasing BCL-2 expression, and Annexin A1 can be considered as a target for treating preeclampsia (88). In addition, injection of heme oxygenase-1 (HO-1) activates the ERK/STAT3 signaling pathway and inhibits the JNK/STAT1 signaling pathway in placental cells, promoting cell survival and alleviating symptoms in a preeclampsia rat model (89). Furthermore, IL-37 inhibits excessive inflammatory response by suppressing the STAT3 signaling pathway in human amniotic cell lines, thus preventing degradation of the extracellular matrix and the occurrence of preterm birth (91). Human placenta-derived mesenchymal stem cells promote migration, repair, and improvement of endometrial glandular cells by activating the JAK2/STAT5 signaling pathway. Additionally, they also stimulate the JNK/Erk1/2-STAT3-VEGF pathway to promote proliferation and migration of human endometrial stromal cells (83). Moreover, in a preeclampsia mouse model, Alpha-1 antitrypsin (AAT) injection inhibits the STAT1 signaling pathway in placental cells, reduces the expression of reactive oxygen species (ROS), increases the expression of superoxide dismutase (SOD), and reduces oxidative stress for the treatment of preeclampsia (84, 85).

**Table 1:** The mechanism for drug treatment of pregnancy-related disorders targeting STATs.

Drug	Species, tissues, or cells	Disease	STATs	Mechanism	Reference
Hyaluronic acid hydrogel-encapsulated human placental mesenchymal stem cells	–	Infertility or RSA	STAT5	Activation of JAK2/STAT5 signaling pathway promotes migration, repair, and improvement of endometrial glandular cells. Activation of JNK/Erk1/2-STAT3-VEGF pathway promotes proliferation and migration of human endometrial stromal cells.	(83)
Alpha-1 antitrypsin	Mouse placental cells	Preeclampsia	STAT1	Inhibition of STAT1 signaling pathway in placental cells, reducing reactive oxygen species expression, increasing superoxide dismutase expression, and decreasing oxidative stress	(84, 85)
Montelukast	Mouse placental cells	Preeclampsia	STAT3	Inhibition of IL-6/JAK2/STAT3 signaling pathway in placental cells	(86)
Lnitroarginine sulfonamide pyridine	Mouse placental cells	Preeclampsia	STAT3	Inhibition of STAT3 activation and reduction of soluble Fms-like tyrosine kinase-1 (sFlt-1) expression in placental tissue	(87)
Silencing Annexin A1	Mouse placental cells	Preeclampsia	STAT3	Inhibition of JAK2/STAT3 signaling pathway, suppressing TNF-α, IL-1β, IL-6, IL-8 expression, reducing inflammation, and inhibiting apoptosis by downregulating Bax and upregulating Bcl-2	(88)
HO-1	–	Preeclampsia	STAT3	Activation of ERK/STAT3 signaling pathway and inhibition of JNK/STAT1 signaling pathway	(89)
Calycosin	β-Islet cells	GDM	STAT3	Inhibition of STAT3 signaling pathway in β-islet cells, promoting cell proliferation and insulin secretion	(90)
IL-37	Mouse placental cells	PTB	STAT3	Inhibition of NF-κB and IL-6/STAT3 signaling pathways in placental cells, suppressing excessive inflammation, ECM remodeling, and apoptosis	(91)
Cho-kyung-jong-ok-tang	Mouse decidua	RSA	STAT6	Activation of STAT6/GATA3 signaling pathway to promote transformation of NK2 cells in mice	(92)
Recombinant adiponectin	Mouse decidua	RSA	STAT5	Regulation of STAT5 signaling pathway to maintain Th17/Treg balance	(93)
Baicalin	Mouse decidua	RSA	STAT5	Inhibition of STAT5/ID2 pathway in conventional dendritic cells of decidua, suppressing their differentiation	(46)
Vitamin D	Human decidua	PTB	STAT1/STAT4, STAT6	Inhibition of STAT1/STAT4 signaling pathway in naïve T cells, suppressing the expression of transcription factor T-bet and inhibiting Th1 differentiation. Activation of STAT6 signaling pathway, promoting transcription factor GATA-3 and Th2 differentiation	(94)
Silymarin	Human decidua	Preeclampsia	STAT3, STAT5	Inhibition of STAT3/RORγt signaling pathway in naïve T cells, suppressing Th17 differentiation. Activation of STAT5/FoxP3 signaling pathway, promoting Treg differentiation	(95)

On the other hand, certain Chinese herbal medicines and related therapeutic methods can intervene and treat pregnancy-related diseases through the STAT signaling pathway. Clinical trials have shown that vitamin D can inhibit the STAT1/STAT4 signaling pathway in human decidual tissue’s naive T cells, suppress the expression of transcription factor T-bet, thereby inhibiting Th1 differentiation, while activating the STAT6 signaling pathway and promoting the transcription factor GATA-3, thus promoting Th2 differentiation (94). In a mouse model of GDM, baicalein inhibits the STAT3 signaling pathway in beta pancreatic cells, thereby promoting cell proliferation and insulin secretion, exerting a therapeutic effect on GDM (90). Curcumin can inhibit the IL-6-mediated STAT3 signaling pathway in mouse decidual cells, suppress the expression of inflammatory factors, and prevent inflammation-induced preterm birth (97). Electroacupuncture treatment activates the LIF/STAT3 signaling pathway in mouse endometrial cells, regulates the surface glycan structure of uterine epithelial cells to improve uterine receptivity, and increase the success rate of pregnancy (98). Studies have also shown that silymarin inhibits the STAT3 signaling pathway in naive T cells, suppresses the expression of RORγ, and inhibits Th17 differentiation, while activating the STAT5 signaling pathway and promoting the expression of FOXP3, which is beneficial for Treg differentiation (95). In a mouse model of miscarriage, recombinant adiponectin regulates STAT5 to induce FOXP3 expression and reduce the expression of RORγ at the maternal-fetal interface, thus promoting Treg differentiation and inhibiting Th17 differentiation, ultimately reducing the rate of miscarriage in mice (93). Additionally, our previous research has shown that baicalin can inhibit the differentiation of conventional DCs in decidual tissue through the STAT5/ID2 pathway, reducing the rate of miscarriage in mice (46). In a mouse model of miscarriage, Cho-kyung-jong-ok-tang (CKJOT) promotes NK2 cell differentiation and improves miscarriage by activating the STAT6/GATA3 signaling pathway (92). Vitamin D and silymarin can prevent preterm birth by regulating the differentiation of T cell subsets.

## 5 Conclusion and perspectives

Pregnancy is a complex process of exogenous embryo implantation in the mother’s body, including endometrialization of the uterus, invasion and migration of trophoblast cells, and maternal-fetal immune tolerance, all of which are necessary for maintaining a normal pregnancy. In this article, we first reviewed the role of STATs signaling pathway in regulating the differentiation and function of endometrial cells, trophoblast cells, and immune cells during normal pregnancy. Moreover, increasing evidence suggests that abnormal expression and function of the STATs signaling pathway are involved in the occurrence and development of various pregnancy-related disorders such as recurrent embryo implantation failure, preeclampsia, preterm birth, recurrent miscarriage, and gestational diabetes. Therefore, targeting the STATs signaling pathway could be an effective approach for preventing and treating pregnancy-related disorders. Currently, several drugs targeting the STATs pathway have been used for the treatment of pregnancy-related diseases. Although the specific mechanisms are not fully understood, it is reasonable to believe that a deeper understanding of the STATs signaling pathway will not only help clarify the occurrence of normal pregnancy but also facilitate the development of targeted therapies for pregnancy-related disorders. However, there is still a long way to go in order to thoroughly understand the functions of STATs in pregnancy and explore immune-based treatments for pregnancy-related disorders based on STATs.

## Author contributions

XL: Conceptualization, Funding acquisition, Supervision, Writing – review & editing. LL: Conceptualization, Data curation, Writing – original draft. ZZ: Funding acquisition, Writing – original draft, Writing – review & editing. HL: Data curation, Writing – original draft. MZ: Data curation, Validation, Writing – original draft. FL: Writing – original draft. CC: Conceptualization, Writing – original draft. YZ: Data curation, Writing – original draft. XZ: Data curation, Writing – original draft. HJ: Conceptualization, Writing – original draft.
